# Supplementary material for: A dual role in regulation and toxicity for the disordered N-terminus of the toxin GraT
Source: Nat Commun. 2019 Feb 27;10:972. doi: 10.1038/s41467-019-08865-z (PMC6393540; doi:10.1038/s41467-019-08865-z)
Supplement: Supplementary file 1 — Supplementary Information [file 41467_2019_8865_MOESM1_ESM.pdf]

Supplementary Information

A dual role in regulation and toxicity for the disordered N-terminus of the toxin GraT

A.Talavera et al.

Supplementary Table 1. X-ray structure determination

	$GraA_2$	$GraA_2$/DNA	GraA2T2
PDB code	6F8H	6F1X	6F8S
Wavelength (Å)	0.978	0.978	0.979
Resolution range (Å)	46.81 - 2.00 (2.07 - 2.00)	43.85 - 3.8 (3.92 - 3.79)	42.18 - 2.5 (2.58 - 2.49)
Space group	$P2_1$	$P\ 3_2$	$P\ 4_1$
Unit cell			
a b c (Å)	62.74 48.95 66.93	105.55 105.55 149.94	56.03 56.03 128.19
α β γ (°)	90 92.62 90	90 90 120	90 90 90
Total reflections	100499 (10135)	96634 (9078)	134815 (12942)
Unique reflections	27427 (2703)	18427 (1797)	13706 (1354)
Multiplicity	3.7 (3.7)	5.2 (5.1)	9.8 (9.6)
Completeness (%)	99.27 (98.11)	99.54 (95.69)	99.87 (98.90)
<I>/σ(I)	19.67 (3.71)	8.84 (0.91)	16.82 (2.05)
R-merge	0.04 (0.34)	0.12 (1.69)	0.09 (0.86)
R-meas	0.05	0.14	0.09
CC1/2	0.99 (0.89)	0.99 (0.39)	0.99 (0.82)
CC*	1 (0.97)	1 (0.74)	1 (0.94)
R-work	0.19 (0.25)	0.24 (0.39)	0.18 (0.27)
R-free	0.22 (0.31)	0.28 (0.42)	0.21 (0.30)
Number of atoms			
macromolecules	2876	4036	2455
ligands	0	0	31
water	244	0	11
Protein residues	377	441	320
RMS(bonds)	0.01	0.006	0.009
RMS(angles)	1.11	1.08	1.18
Ramachandran (%)			
Favored	100	95	98
Allowed	0	5	2
Outliers	0	0	0
Clashscore	3.49	10.97	6.70
B-factors (Å^2)			
From Wilson Plot	32.11	156.43	59.46
All atoms	37.30	177.50	66.20
Protein atoms	36.80	177.50	65.90
Water atoms	43.10		90.40
Other atoms			68.20

Supplementary Table 2. Bacterial strains, plasmids and oligonucleotides

**Strain or plasmid**	**Genotype or characteristics**	**Source or reference**
E. coli		
DH5α	*supE44* Δ*lacU169* (α80 *lacZ*ΔM15) *recA1 endA1 hsdR17 thi-1 gyrA96 relA1*	[2]
BL21(DE3)	*hsdS gal* (λc*Its*857 *ind*1 *S*am7 *nin*5 *lac*UV5-T7 gene *1*)	[3]
BW25113	Δ(*araD-araB*)*567*, Δ*lacZ4787*(*::rrnB-3*), Δ(*rhaD-rhaB*)*568, hsdR514*	[4]
MG1655 Δ10 TA	MG1655 Δ*mazF,* Δ*chpB,* Δ*relBE,* Δ(*dinJ-yafQ*), Δ(*yefM-yoeB*), Δ*higBA,* Δ(*prlF-yhaV*), Δ*yafNO,* Δ*mqsRA,* Δ*hicAB*	[5]
P. putida		
PaW85	Wild-type, isogenic to KT2440	[6]
ΔgraA	*graA* is deleted from PaW85	[7]
Δ22graT	Nucleotides coding for amino acids 2-22 are deleted from *graT* in PaW85	This study
ΔgraAΔ22graT	Nucleotides coding for amino acids 2-22 are deleted from *graT* in ΔgraA	This study
Plasmids		
pET11c	Protein expression vector (Ap^r)	Stratagene
pET-hisA	Plasmid for expression of GraA with N-terminal His$_6$-tag (Ap^r)	[8]
pET-hisTA	Plasmid for expression of GraT with N-terminal His$_6$-tag and untagged GraA (Ap^r)	[7]
pET-Δ22graTA-his	Plasmid for expression of N-terminally truncated GraT and GraA with C-terminal His$_6$-tag (Ap^r)	This study
pET-Δ22graTA	Plasmid for expression of N-terminally truncated GraT and tagless GraA (Ap^r)	This study
pET-hisTEVgraTA	Plasmid for expression of GraT with N-terminal His$_6$ and TEV protease tags and tagless GraA (Ap^r)	This study
pET21b-GraTΔ92-TEV-his[b]	Plasmid for expression of GraT *wi*th C-terminal His92 deleted followed by TEV protease site and His$_6$-tag	This study
pBRlacItac	Expression vector containing P$_{tac}$ promoter and *lacI*^{q} repressor in pBR322 (Ap^r)	[9]
pBBR1-MCS-5	Broad-host-range cloning vector (Gm^r)	[10]
pBBR(Gm)lacItac	Expression vector containing P$_{tac}$ promoter and *lacI*^{q} repressor in pBBR1-MCS-5 (Gm^r)	This study
pET-A	Plasmid for overexpression of tagless GraA (Ap^r)	[7]
pBBRlacItac-graA	Plasmid for overexpression of tagless GraA (Gm^r)	This study
pET-graTA	Plasmid for overexpression of tagless GraTA (Ap^r)	[11]
pBBRlacItac-graTA	Plasmid for overexpression of tagless GraTA (Gm^r)	This study
pBBRlacItac-Δ22graTA	Plasmid for overexpression of tagless Δ22GraTA (Gm^r)	This study
pBBRlacItac-Δ22graT	Plasmid for overexpression of tagless Δ22GraT (Gm^r)	This study
p9TT1586	Plasmid containing *graTA* promoter fused with *lacZ* (Ap^r Cm^r)	[7]
pEMG	Suicide plasmid containing *lacZ*α with two flanking I-SceI sites (Km^r)	[1]
pEMG-Δ22graTA	pEMG with a PCR-designed 1.18 kb EcoRI-BamHI insert for deleting N-terminus of *graT* in wild-type (Km^r)	This study
pEMG-Δ22graTΔA	pEMG with a PCR-designed 913 bb EcoRI-BamHI insert for deleting N-terminus of *graT* in ΔgraA (Km^r)	This study
pSW(I-SceI)	Plasmid for I-SceI expression (Ap^r)	[12]
**Oligo Name**	**Sequence (5'-3')[a]**	**Use**
GraT_22_Nde	CAG<u>CATATG</u>TCAGATATCAAGTCAGTCGC	construction of pET-Δ22graTA-his and pET-Δ22graTA
A-his	CGT<u>GGATCC</u>TTAGTGATGGTGGTGATGATGAGCGACCAGACGCTGGACCGA	construction of pET-Δ22graTA-his
1585Bam	AT<u>GGATCC</u>GTTTTTCGATGTCAGT	construction of pET-Δ22graTA, pET-hisTEVgraTA and pEMG-

		Δ22graTA
hisTEV-graT	CTCCATATGCATCACCACCACCATCACGAGAACCTGTATTTCCAAGGCATTCGAAGCTTTAGCT GTGC	construction of pET-hisTEVgraTA
pETSal	TTTGTCGACTTTAAGAAGGAGATATACAT	construction of pBBRlacItac-graA, pBBRlacItac-graTA, pBBRlacItac-Δ22graTA, and pBBRlacItac-Δ22graT
1585Acc	ATGGTACCGTTTTTCGATGTCAGTCG	construction of pBBRlacItac-graA, pBBRlacItac-graTA, and pBBRlacItac-Δ22graTA
1586Bam	CGGGATCCGTTCTTGAGCATGATGC	construction of pBBRlacItac-Δ22graT
T_N22delpikk	CGTTAAGCATTCAGCTCATGTCAGATATCAAGTCAGTCG	construction of pEMG-Δ22graTA
1586ATG	CATGAGCTGAATGCTTAACG	construction of pEMG-Δ22graTA
TAdelEco	GGGGAATTCGGCAGCAACCAGTGGAT	construction of pEMG-Δ22graTA
lpp_ec	TTACTTGCGGTATTTAGTAGCC	primer extension of *lpp* mRNA, sequencing
lpp_ec_ees	ACGTTGTAGTTATGGTTT	generation of sequencing template

[a] The sites for restriction enzymes are underlined.

[b] GraTΔ92-TEV-his was chemically synthetized, encoding for the following protein sequence:

MIRSFSCADTEALFTTGKTRRGSDIRSFSCADTEALFTTGKTRRGSDIKSVAERKLAMLDAATELRDLRSPPGNRLESLSGNRADQHS IRVNDQWRLCFTWTEHGPVNVEIVDYENLYFQGSAGHHHHHH

Supplementary Table 3: Crystallization results

	Crystallization conditions Condition
HisGraA_2	0.1 M KCl, 0.1 M HEPES pH 7.5, 15%(w/v) PEG 6000
hisGraT_2A_2	0.8 M Li2SO4, 0.1 M sodium acetate trihydrate pH 4.0, 4%(v/v) PEG 200
hisGraA_2/*graTA* GraA_2	0.2 M lithium sulfate, 0.1 M sodium acetate pH 4.5, 50% PEG 400

Supplementary Table 4: Sample details for SAXS experiments

	GraTA	GraT
SASBDB.org accession code	SASDE58	SASDE48
Organism	*Pseudomonas putida*	*Pseudomonas putida*
Source	*E. coli*	*E. coli*
UniProt ID	Q88MI5 - Q88MI6	Q88MI5
Ext. Coeff (A280, 0.1 % (*w/v*))	0.849	1.01
MW (Da)	21334.25	11104.44
SEC-SAXS column	Shodex KW404-4F 500 kDa	
Loading concentration (mg/ml)	10	12.5
Injection volume (μl)	40	40
Flow rate (ml/min)	0.2	0.2
Solvent	50 mM Tris pH 8.0, 150 mM NaCl, 2 mM TCEP	

Supplementary Table 5: SAXS data collection parameters.

	GraTA	GraT
Instrument	Eiger-4M	Eiger-4M
Wavelength (Å)	1.03320054431	1.0331619082
Beam size (μM)		
Camera length (m)	1.99	1.499
q measurement range ($Å^{-1}$)	0.0036 - 0.5596	0.0048 - 0.81665
Absolute scaling method		
Normalization		
Exposure time (s)	0.99	0.99
Sample configuration	SEC-SAXS	SEC-SAXS
Sample temperature (ºC)	25	25

Supplementary Table 6: Software for SAXS data reduction, analysis and interpretation.

SAXS data reduction	Foxtrot, ScÅtter 3.1
Extinction coefficient estimate	ProtParam
Basic analyses: Guinier, *P(r)*, V_P	Primus from ATSAS 2.8.0
Shape/bead modeling	DAMIF via ATSAS
Atomic structure modeling	FoXS, MultiFoXS
Missing sequence modeling	Modeller
3D graphics model representation	Pymol

Supplementary Table 7: SAXS Structural parameters.

	GraTA	GraT
**Guinier analysis**		
I(0) (cm^{-1})	0.04879 ± 0.000028	0.01444 ± 0.000015
R_g (Å)	23.49 ± 0.05	15.18 ± 0.61
q_{min} ($Å^{-1}$)	0.0137	0.0274
qRg max	1.29526	1.299
Coefficient correlation, R^2	0.99	0.96
M (Da) from I(0) (ratio to predicted)	40991 (0.93)	9548 (0.85)
***P(r)* analysis**		
I(0)	0.002153 ± 0.000014	0.01444 ± 0.000022
R_g (Å)	22.9 ± 0.2	16.07 ± 0.82
d_{max} (Å)	79.06	70.4
q range ($Å^{-1}$)	0.0137 - 0.3403	0.0274 - 0.5270
χ^2 (total estimate from GNOM)	0.99	0.96
M from I(0) (ratio to predicted)	40991 (0.93)	9548 (0.85)
Porod Volume ($Å^3$) (ration V_P/calculated M)	60700 (1.48)	10058 (0.91)
M using Fisher method (ratio of M to expected)	43730 (1.02)	10200 (0.92)

Supplementary Table 8: SAXS Atomistic modeling.

	GraTA		GraT	
Crystal Structures (PDB codes)	6F8S		6F8S	
	Disordered ensemble N-terminus	HigB-like N-terminus	Disordered ensemble N-terminus	HigB-like N-terminus
MultiFoxs/Foxs				
χ^2	1.42	10.16	3.15	8.9
Predicted R_g (Å)	21.5 - 23.5	21.83	14.1 – 23.7	13.9
c_1, c_2	1.01, -0.3	0.99, 0.45	1.01, 0.26	1.02 - 0.54

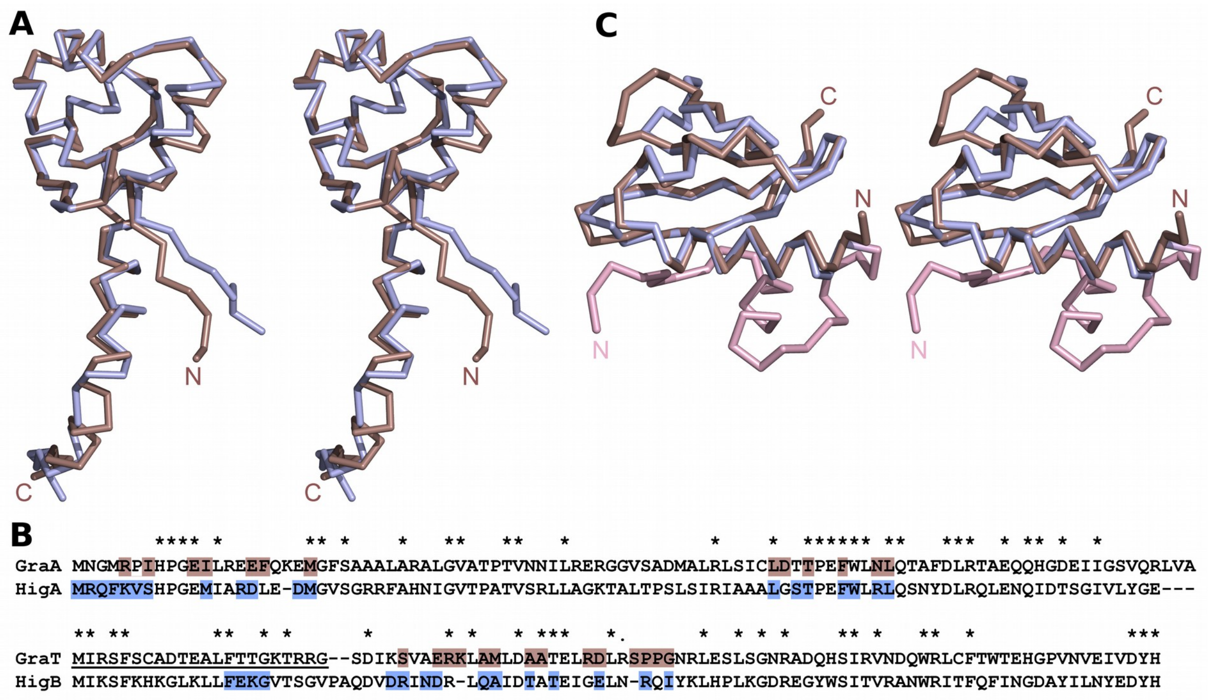

Supplementary Figure 1. **Comparison of GraA and GraT with *P. vulgaris* HigA and HigB. A.** Stereo view of the superposition of the *P. putida* GraA monomer (brown Cα trace) on the *P. vulgaris* HigA monomer (blue Cα trace). The N- and C-termini of GraA are indicated. B. Structure-based sequence alignments of GraA with HigA and GraT with HigB. The disordered N-terminus of GraT (sequence is underlined) was aligned manually with the ordered N-terminal sequence of HigB. Identical residues are indicated with a star. Residues involved in the toxin-antitoxin interface are highlighted: brown for GraA/GraT and blue for HigA/HigB. C. Stereo view of the superposition of the *P. putida* GraT monomer (brown Cα trace) on the *P. vulgaris* HigB monomer (blue Cα trace). The ordered N-terminal 25 amino acids of HigB are highlighted in pink. N-and C-termini of GraT are indicated in brown.

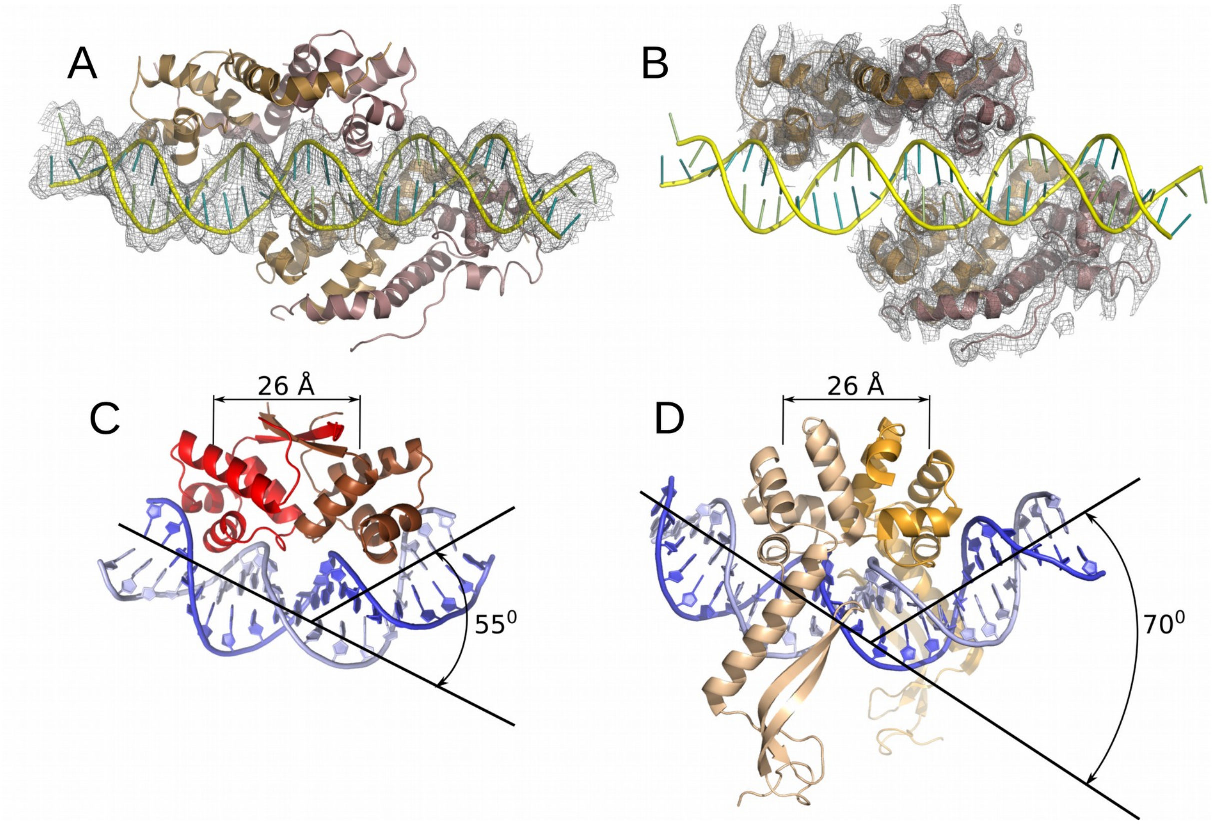

Supplementary Figure 2. **GraA-DNA complex and operator complexes of HipB and MqsA. A.** 2Fo – Fc electron density map at 1σ contour level of the DNA component in the GraA-DNA complex. **B.** Similar 2Fo – Fc electron density map at 1σ contour level for the $GraA_2$ component in the GraA-DNA complex. **C.** Cartoon representation of the operator complex of *E. coli* HipB. The distance between the two HTH motifs of the HipB dimer (26 Å) as well as the bending of the DNA by 55° are indicated. **D.** Cartoon representation of the operator complex of *E. coli* MqsA. The bending of the DNA by 70° is indicated, as is the separation of 26 Å between the two HTH motifs.

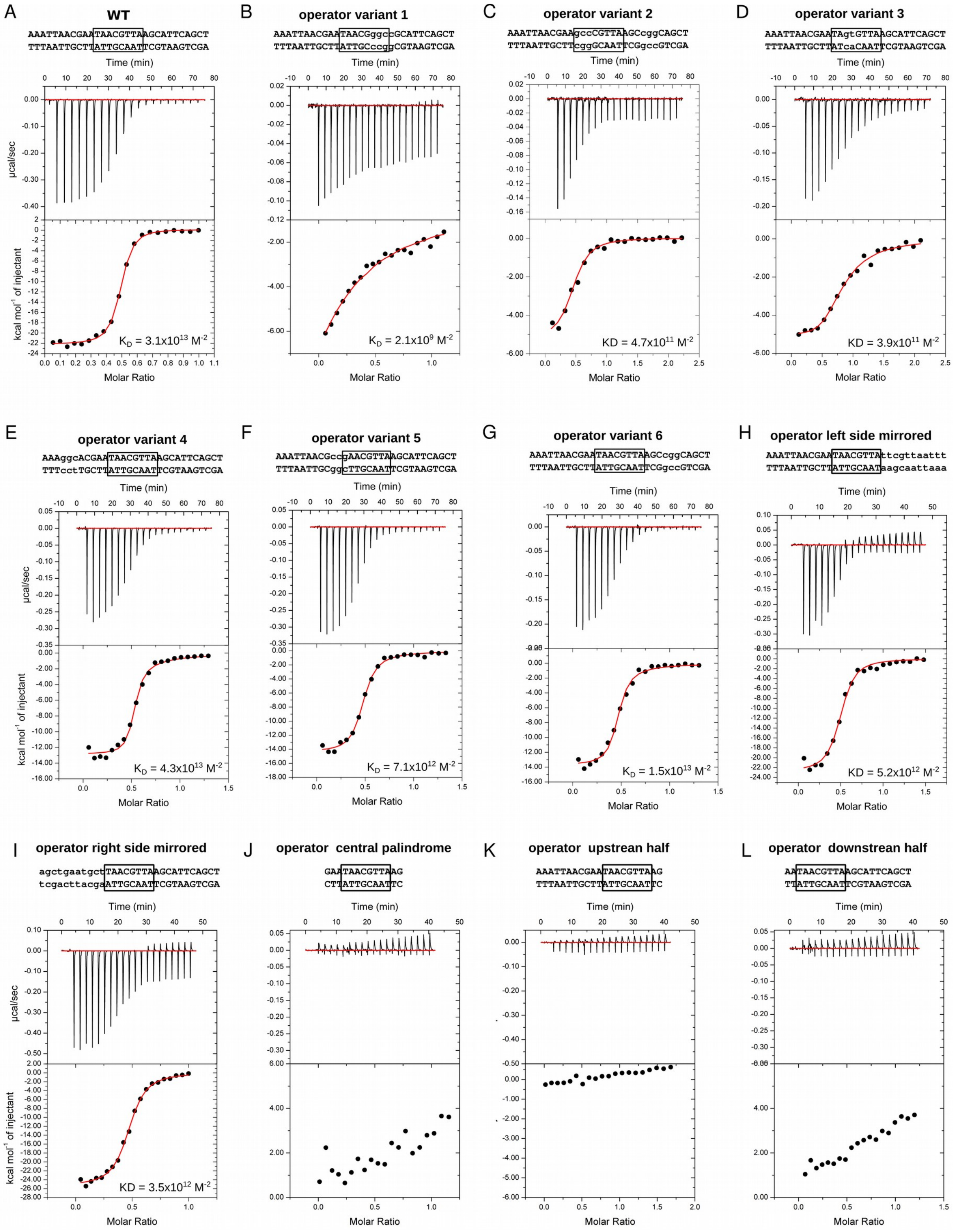

Supplementary Figure 3. **Operator binding by GraA$_2$ *in vitro*.** ITC experiments of different variants of the *graTA* operator (as in Table 1) titrated into GraA$_2$ at 25°C. Wild-type sequences are given in capital letters, while mutated base positions are given by small letters. The box represents the position of the central palindrome. For each panel, the upper part shows the heat generated by the individual injections while the lower part provides the corresponding fitted titration isotherm at 25°C. Panel A shows the titration of the wild-type operator, and panels from B to L show the titration with different variants of the operator. B shows variant 1, C variant 2, D variant 3, E variant 4, F variant 5, G variant 6, H left side mirrored, I right side mirrored, J central palindrome, K upstream half, L downstream half.

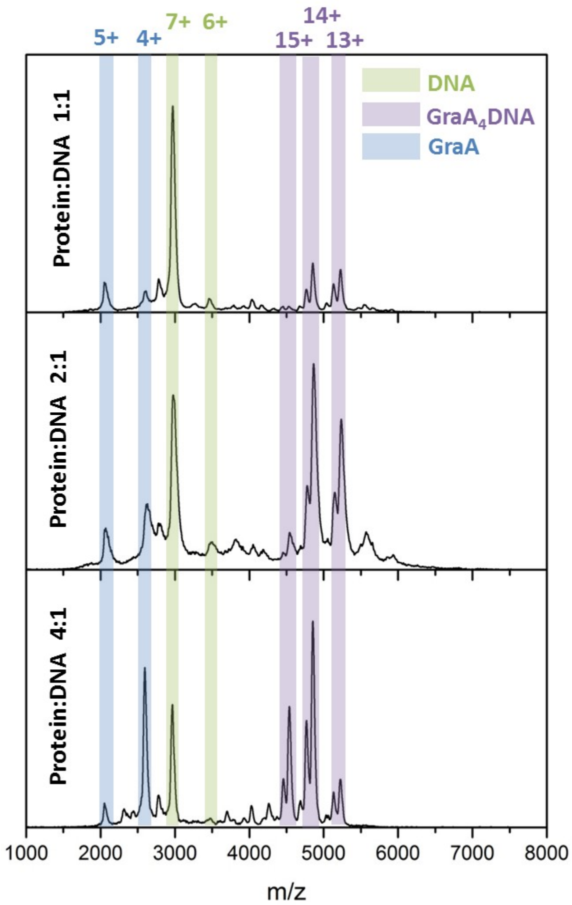

Supplementary Figure 4. **Native mass spectrometry of GraA-operator complexes.** $GraA_2$ and operator were mixed at different protein:DNA ratios in 40 mM aqueous ammonium acetate at pH 7. Peaks corresponding to GraA monomer, DNA duplex and the $GraA_4DNA$ complex are indicated. No peaks are observed for a $GraA_2DNA$ species.

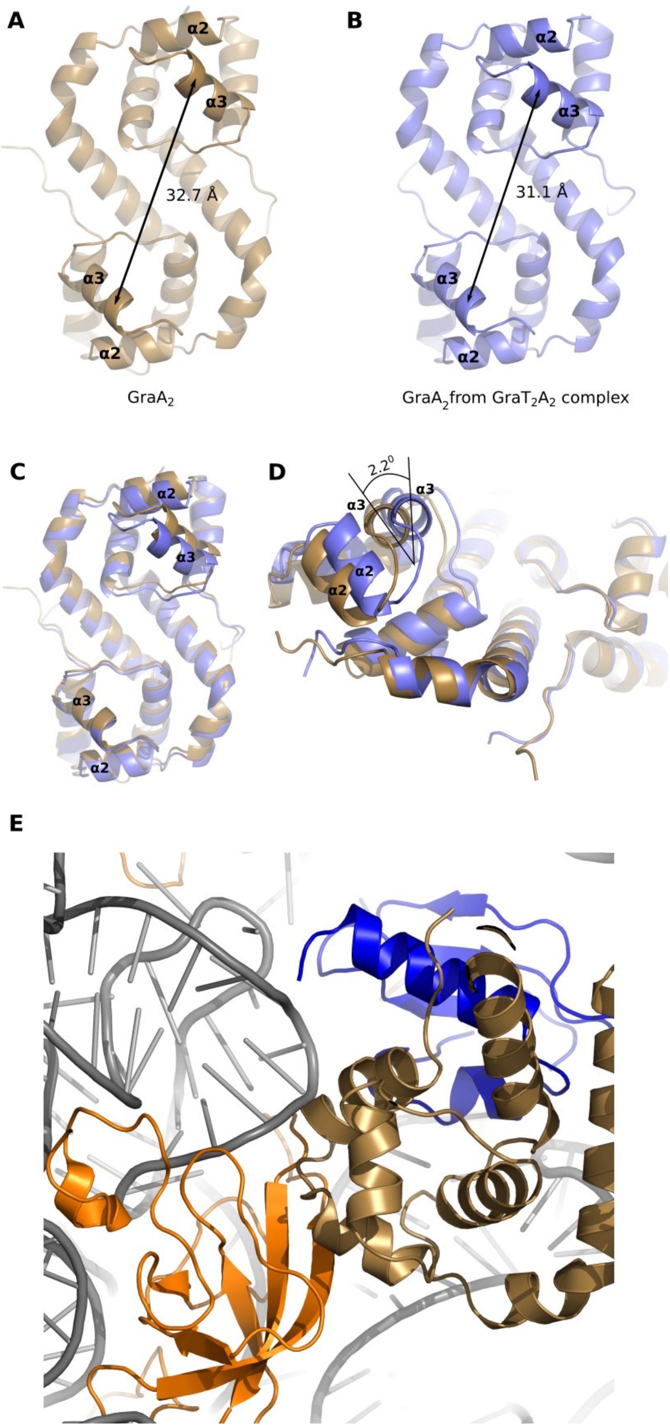

Supplementary Figure 5. Comparison of the structures of $GraA_2$ in its free state and in the $GraT_2A_2$ complex. A. Ribbon representation of the $GraA_2$ dimer. The distance between the two recognition helices α3 of the HTH motif is indicated. **B.** Equivalent view of the $GraA_2$ dimer as present in the $GraA_2T_2$ complex. **C.** Superposition of the two $GraA_2$ dimers using a single monomer as reference. **D.** Zoom-in of panel C. to show the relative rotation of 2.2° of one GraA monomer when comparing the free and GraT-bound states. **E.** Superimposition of GraT from $GraA_2T_2$ complex into HigB bound to the ribosome (PDB entry 4W4G). The ribosome is shown in gray (RNA) and orange (ribosomal proteins). GraA is shown in sand color and GraT in blue.

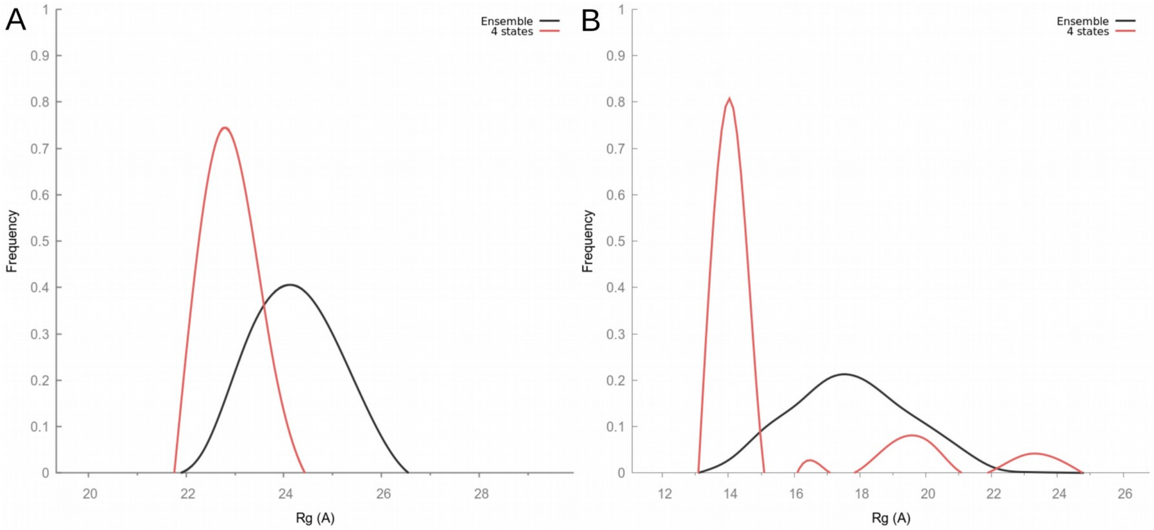

Supplementary Figure 6. MultiFoxs multi-state histograms. Rg distribution of the different multistate models. Black line is the Rg distribution of the initial 10 000 generated conformations, and red line represents the distributions for the four-states model. **A)** GraTA complex, **B)** GraT alone.

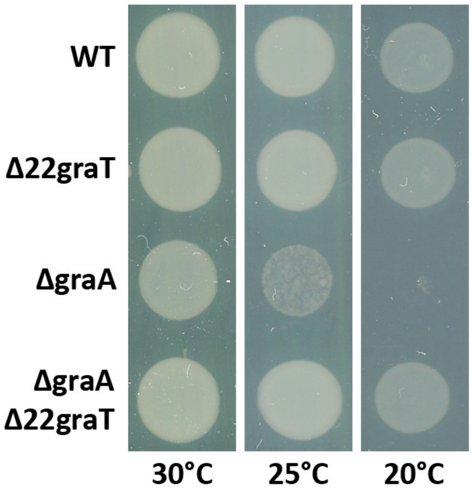

Supplementary Figure 7. The intrinsically disordered N-terminus of GraT is required for toxicity *in vivo*. Antitoxin deletion from *P. putida* chromosome (strain ΔgraA) results in GraT-mediated cold-sensitive growth defect, which is abolished by truncation of GraT at its N-terminus (strain ΔgraAΔ22graT). The *P. putida* wild-type PaW85 (wt), ΔgraA and their Δ22*graT* derivative strains Δ22graT and ΔgraAΔ22graT were grown on LB solid medium at 30 °C, 25 °C and 20 °C for 24 hours. Approximately 10^5 cells were inoculated per spot.

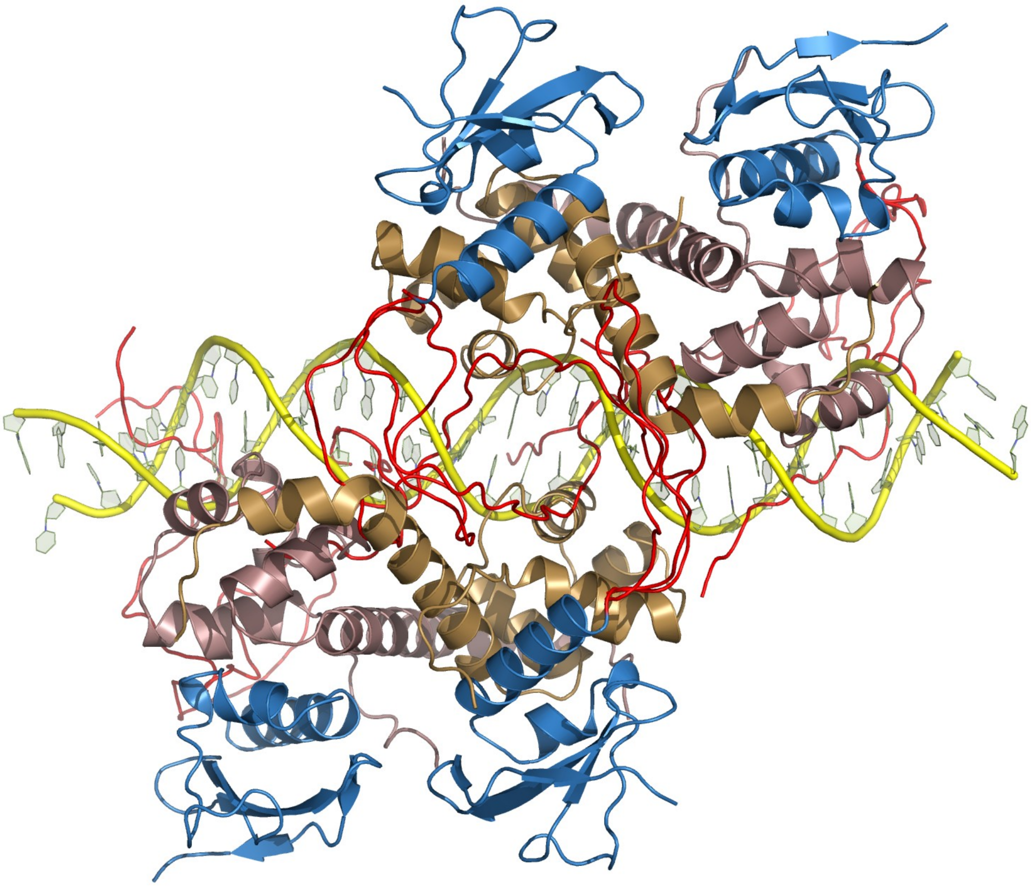

Supplementary Figure 8. Model for the hypothetical $GraT_2A_2$-operator complex. The model was generated by superimposing the GraT-$GraA_2$-GraT complex upon the $GraA_2$ moieties of the $(GraTA)_2$-operator complex. No steric clash is induced by the presence of the globular part of GraT that could be invoked as an explanation why this complex only binds very weakly to the operator. The disordered N-termini of GraT are represented here as a single random but rather extended conformation (colored red), to illustrate that on average, this segment points towards the DNA.

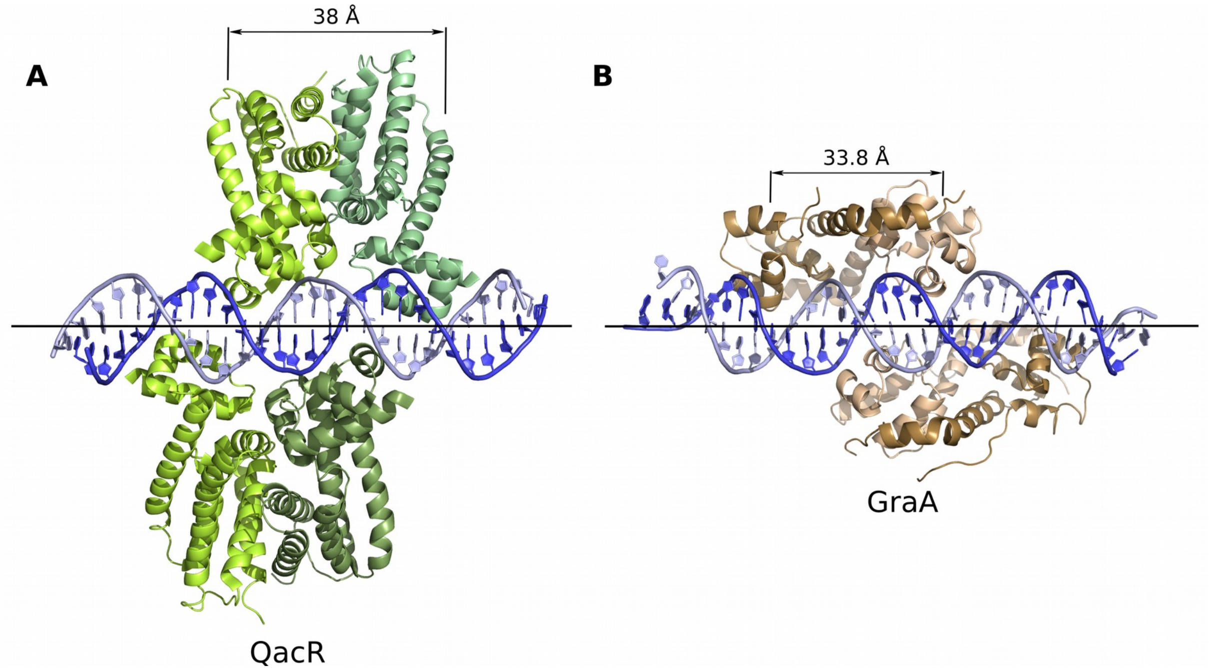

Supplementary Figure 9. **Comparison between the operator complexes of GraA and QacR. A.** Operator complex for QacR, a TetR family member from *Staphylococcus aureus*. Both QacR dimers are bound on opposite sides of the operator. **B.** Operator complex for GraA. The architectural similarities between the GraA and QacR complexes are striking.

Supplementary Methods

Construction of plasmids and strains

Plasmids, strains and oligonucleotides used in PCR amplifications are listed in Supplementary Table 2. For construction of plasmid pET-Δ22graTA-his, the modified *graTA* operon was amplified with the aid of oligonucleotides GraT_22_Nde and A-his, the obtained PCR product was treated with NdeI and BamHI and cloned into the corresponding sites in pET11c. Plasmid pET-Δ22graTA, encoding a truncated Δ22GraT and tagless GraA, was constructed similarly, except that primers GraT_22_Nde and 1585Bam were used for PCR amplification. For construction of pET-hisTEVgraTA, the His_6-tag and TEV protease cleavage site were fused to the N-terminus of GraT by PCR and the modified *graTA* operon was amplified with the aid of oligonucleotides hisTEV-graT and 1585Bam. The NdeI-BamHI-treated PCR fragment was cloned into NdeI-BamHI-cleaved pET11c.

For construction of a expression vector pBBR(Gm)lacItac, the 1.9 kb BamHI-SalI fragment containing the *lacItac* cassette was cut from pBRlacItac and inserted into the corresponding sites in pBBR1-MCS-5. In order to construct the GraA, GraTA and Δ22graTA expression plasmids pBBRlacItac-graA, pBBRlacItac-graTA, and pBBRlacItac-Δ22graTA, the *graA*, *graTA* and Δ22*graTA* genes together with the SD region of the pET plasmid were PCR-amplified from pET-A, pET-graTA and pET-Δ22graTA, respectively, using oligonucleotides 1585Acc and pETSal. PCR fragments were treated with KpnI and SalI and ligated into the KpnI-SalI-opened pBBR(Gm)lacItac. For construction of pBBRlacItac-Δ22graT the Δ22*graT* Δne together with the SD region of the pET plasmid was PCR-amplified from pET-Δ22graTA using oligonucleotides 1586Bam and pETSal and cloned into pBBR(Gm)lacItac.

The Δ22graT derivatives of *P. putida* wild-type and ΔgraA strains were generated by integrating/excising the delivery plasmids pEMG-Δ22graTA and pEMG-Δ22graTΔA, respectively, with the procedure described elsewhere [1]. For construction of these plasmids, the upstream and downstream regions of wanted deletion in *graT* were amplified with primer pairs 1586ATG/TAdelEco and 1585Bam/T_N22delpikk, respectively. *P. putida* wild-type or ΔgraA strains were used as templates in amplification of Δ22graTA and Δ22graTΔA fragments, respectively. The obtained PCR products were then joined into one fragment by overlap extension PCR using oligos TAdelEco and 1585Bam. The 1.18 kb Δ22graTA and 913 bp Δ22graTΔA fragments were cloned into EcoRI-BamHI-opened plasmid pEMG. Plasmid pEMG-Δ22graTA was then delivered to *P. putida* wild-type and pEMG-Δ22graTΔA to ΔgraA strain by electroporation and kanamycin-resistant colonies carrying a cointegrate in the chromosome were isolated on kanamycin plates. For resolving the cointegrates, the selected

clones were electroporated with plasmid pSW(I-SceI) enabling expression of I-SceI nuclease. After I-SceI was induced overnight with 1.5 mM 3-methylbenzoate, the kanamycin-sensitive colonies were selected, and the deletion of the truncation of the *graT* was verified by PCR and sequencing. Plasmid pSW(I-SceI) was eliminated from the deletion strains by growing them overnight in LB medium without antibiotics.

Supplementary references:

1. Martinez-Garcia, E., and de Lorenzo, V. (2011). Engineering multiple genomic deletions in Gram-negative bacteria: analysis of the multi-resistant antibiotic profile of Pseudomonas putida KT2440. Environmental microbiology 13, 2702-2716.
2. Hanahan, D., and Meselson, M. (1983). Plasmid screening at high colony density. Methods in enzymology 100, 333-342.
3. Studier, F.W., and Moffatt, B.A. (1986). Use of bacteriophage T7 RNA polymerase to direct selective high-level expression of cloned genes. Journal of molecular biology 189, 113-130.
4. Datsenko, K.A., and Wanner, B.L. (2000). One-step inactivation of chromosomal genes in Escherichia coli K-12 using PCR products. Proceedings of the National Academy of Sciences of the United States of America 97, 6640-6645.
5. Maisonneuve, E., Shakespeare, L.J., Jorgensen, M.G., and Gerdes, K. (2011). Bacterial persistence by RNA endonucleases. Proceedings of the National Academy of Sciences of the United States of America 108, 13206-13211.
6. Bayley, S.A., Duggleby, C.J., Worsey, M.J., Williams, P.A., Hardy, K.G., and Broda, P. (1977). Two modes of loss of the Tol function from Pseudomonas putida mt-2. Molecular & general genetics : MGG 154, 203-204.
7. Tamman, H., Ainelo, A., Ainsaar, K., and Hõrak, R. (2014). A Moderate Toxin, GraT, Modulates Growth Rate and Stress Tolerance of Pseudomonas putida. Journal of bacteriology 196, 157-169.
8. Tamman, H., Ainelo, A., Tagel, M., and Hõrak, R. (2016). Stability of the GraA antitoxin depends on the growth phase, ATP level, and global regulator MexT. Journal of bacteriology 198, 787-796.
9. Ojangu, E.L., Tover, A., Teras, R., and Kivisaar, M. (2000). Effects of combination of different -10 hexamers and downstream sequences on stationary-phase-specific sigma factor sigma(S)-dependent transcription in Pseudomonas putida. Journal of bacteriology 182, 6707-6713.
10. Kovach, M.E., Elzer, P.H., Hill, D.S., Robertson, G.T., Farris, M.A., Roop, R.M., 2nd, and Peterson, K.M. (1995). Four new derivatives of the broad-host-range cloning vector pBBR1MCS, carrying different antibiotic-resistance cassettes. Gene 166, 175-176.
11. Ainelo, A., Tamman, H., Leppik, M., Remme, J., and Hõrak, R. (2016). The toxin GraT inhibits ribosome biogenesis. Molecular microbiology 100, 719-734.
12. Wong, S.M., and Mekalanos, J.J. (2000). Genetic footprinting with mariner-based transposition in Pseudomonas aeruginosa. Proceedings of the National Academy of Sciences of the United States of America 97, 10191-10196.
